# Discovery of half‐curcumin‐based chemiluminescence probes and their applications in detecting quasi‐stable oxidized proteins

**DOI:** 10.1002/alz.090104

**Published:** 2025-01-09

**Authors:** Chongzhao Ran, Jun Yang

**Affiliations:** ^1^ Massachusetts General Hospital and Harvard Medical School, Boston, MA USA

## Abstract

**Background:**

Oxidative stress, which refers to an imbalance between the production of reactive oxygen/nitrogen species (ROS/RNS) and the body's antioxidant defense mechanisms, is implicated in the pathogenesis of various chronic diseases by promoting cellular damage, inflammation, and dysfunction. Numerous methods have been reported for detecting ROS/RNS in vitro and in vivo; however, detecting methods for the secondary products of the ROS/RNS reactions, particularly quasi‐stable oxidized products, have been much less explored.

**Method:**

In this report, we discovered that acetylacetone, a core moiety of curcumins, is a new scaffold for generating chemiluminescence in the presence of oxidants. With this new scaffold, we designed a series of half‐curcumins CRANAD‐Xs and demonstrated that CRANAD‐164 could be used to detect quasi‐stable oxidized proteins (QSOP) in patient serum samples and for in vivo imaging in animal models.

**Result:**

We found that the QSOP levels were much higher in the disease serum samples, including Alzheimer’s disease, diabetes, multiple sclerosis, and pulmonary fibrosis, compared to the samples from healthy controls. Moreover, our results revealed that the sera chemiluminescence intensities were higher in aged healthy controls (60‐year old) compared to young healthy subjects (20‐year old), suggesting that CRANAD‐164 can be used to monitor the increase of QSOP in the course of aging.

**Conclusion:**

Our results suggest that acetylacetone‐based chemiluminescence probes can potentially reflect the health status of a patient’s serum (blood) via in vitro diagnosis. In addition, the probes’ in vivo imaging capacity can be harnessed to facilitate fundamental studies and drug discovery of inflammatory diseases.

